# Supplementary material for: Strawberry FaSnRK1α Regulates Anaerobic Respiratory Metabolism under Waterlogging
Source: Int J Mol Sci. 2022 Apr 28;23(9):4914. doi: 10.3390/ijms23094914 (PMC9101944; doi:10.3390/ijms23094914)
Supplement: Supplementary file 1 [file ijms-23-04914-s001.zip › Supplementary Materials/Figure S1.pdf]

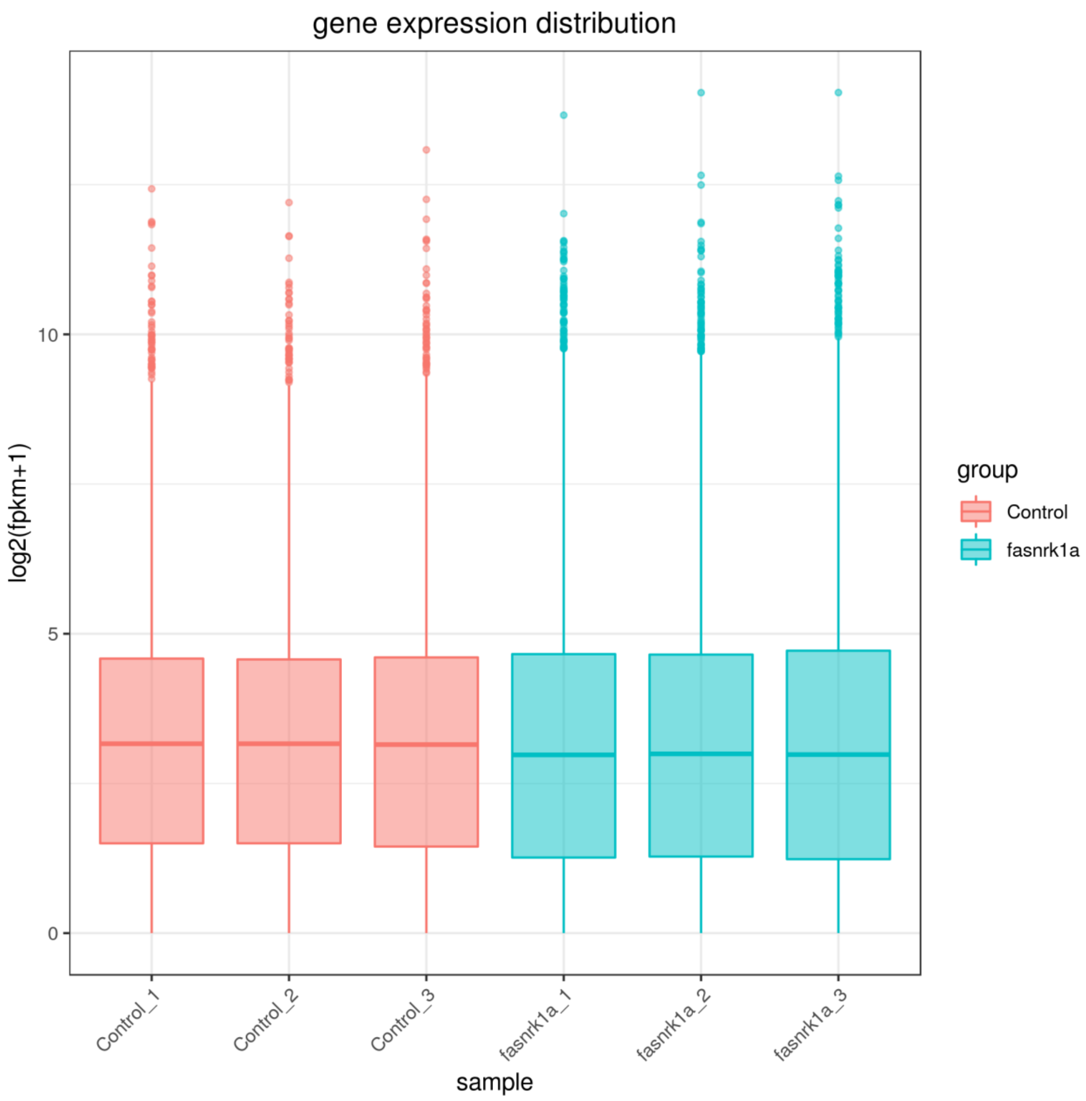

Figure S1: Scatter plot of FPKM values between replicates or genotypes. The $\log_{10}$ transformed values of replicates of control and *fasnrk1a* are plotted.
