# Supplementary material for: Strawberry FaSnRK1α Regulates Anaerobic Respiratory Metabolism under Waterlogging
Source: Int J Mol Sci. 2022 Apr 28;23(9):4914. doi: 10.3390/ijms23094914 (PMC9101944; doi:10.3390/ijms23094914)
Supplement: Supplementary file 1 [file ijms-23-04914-s001.zip › Supplementary Materials/Table S1.pdf]

Table S1:Mapping statistics of RNA-seq reads

sample	total_reads	clean_reads	Q30	GC_pct	total_map	unique_map	multi_map	positive_map
Control_1	42193968	42193968	93.51	45.45	32930768(78.05%)	32338392(76.64%)	592376(1.4%)	16142569(38.26%)
Control_2	44863468	44863468	93.79	45.50	35211854(78.49%)	34587961(77.1%)	623893(1.39%)	17265210(38.48%)
Control_3	44717728	44717728	93.58	46.46	35989412(80.48%)	35325238(79.0%)	664174(1.49%)	17641183(39.45%)
fasnrk1a_1	41959212	41959212	93.49	46.78	33613034(80.11%)	33008630(78.67%)	604404(1.44%)	16471397(39.26%)
fasnrk1a_2	43535856	43535856	93.44	46.79	34226530(78.62%)	33583082(77.14%)	643448(1.48%)	16753692(38.48%)
fasnrk1a_3	42584920	42584920	93.29	46.94	33934523(79.69%)	33179661(77.91%)	754862(1.77%)	16554422(38.87%)
