# Supplementary material for: Strawberry FaSnRK1α Regulates Anaerobic Respiratory Metabolism under Waterlogging
Source: Int J Mol Sci. 2022 Apr 28;23(9):4914. doi: 10.3390/ijms23094914 (PMC9101944; doi:10.3390/ijms23094914)
Supplement: Supplementary file 1 [file ijms-23-04914-s001.zip › Supplementary Materials/Table S5.pdf]

Table S5. Specific primers used for amplification genes

Names of genes	primer (5' to 3')
FaSnRK1α	F：TGGCGCGCCACTAGTGGATCCATGGATGGAGCAATTGGCC
(pCAMIBIA1302)	R：CATGGTACCCTCGAGGTCGACAAGAACACGAAGCTGTGCAAGG
FaSnRK1α	F：GTGAGTAAGGTTACCGAATTCCTACTTATTATTGGACAACCGGTTTC
(TRV2-1)	R：CGTGAGCTCGGTACCGGATCCGGGAACTGCTGTCTAAAAGGTGA
FaSnRK1α	F：GTGAGTAAGGTTACCGAATTCGAAAATTAAGGGCGGGATATACAC
(TRV2-2)	R：CGTGAGCTCGGTACCGGATCCGGGGCACAGCTAAGTAACGAGG
FaSnRK1α	F：GTGAGTAAGGTTACCGAATTCGGCCGTGGAGGCAGCAGC
(TRV2-3)	R：CGTGAGCTCGGTACCGGATCCTGCGCCGGTTAAGAATCTTG
