# Supplementary material for: Strawberry FaSnRK1α Regulates Anaerobic Respiratory Metabolism under Waterlogging
Source: Int J Mol Sci. 2022 Apr 28;23(9):4914. doi: 10.3390/ijms23094914 (PMC9101944; doi:10.3390/ijms23094914)
Supplement: Supplementary file 1 [file ijms-23-04914-s001.zip › Supplementary Materials/Table S6.pdf]

Table S6. Specific primers used for real-time PCR analysis

Gene	Forward sequence (5’-3’)	Reverse sequence (5’-3’)
FaSnRK1α	GCATCCTCACATTATACGACTCTA	TCCAGACTTCACATACTCCATAAC
FaACTIN	TGGGTTTGCTGGAGATGAT	CAGTAGGAGAACTGGGTGC
FaPDC1	GATTTGTATCGTTGGCGGGC	GGGTCTCTACCAAACGTGGG
FaADH1	TAACTACAAGCCCCGCACTG	GGATGATGCACCGGATTCCT
FaHRE2	GGCGGTGATAGAATCGCTGA	ATGGGTTGGGGTGTTTTGGT
FaRAP2.3	GTCTGGCTCGGAACCTTCAA	CATGGTCGTGATCAGCCGTA
FaRAP2.12	TACAGGGGAATTCGTCAGCG	TTCTACGTGCCTCAGCATCG
